# High-throughput SNP-genotyping analysis of the relationships among Ponto-Caspian sturgeon species

**DOI:** 10.1002/ece3.659

**Published:** 2013-07-01

**Authors:** Sergey M Rastorguev, Artem V Nedoluzhko, Alexander M Mazur, Natalia M Gruzdeva, Alexander A Volkov, Anna E Barmintseva, Nikolai S Mugue, Egor B Prokhortchouk

**Affiliations:** 1 National Research Center “Kurchatov Institute”Kurchatov sq. 1, 123182, Moscow, Russia; 2 Russian Federal Research Institute of Fisheries and Oceanography (VNIRO)V. Krasnoselskaya str. 17, 107140, Moscow, Russia; 3 Center of Bioengineering, Russian Academy of Sciences60-letiya Oktyabrya av. 7-1, 117312, Moscow, Russia

**Keywords:** Genotyping, glaciation, population genetics, SNP, species relationships, sturgeon

## Abstract

Legally certified sturgeon fisheries require population protection and conservation methods, including DNA tests to identify the source of valuable sturgeon roe. However, the available genetic data are insufficient to distinguish between different sturgeon populations, and are even unable to distinguish between some species. We performed high-throughput single-nucleotide polymorphism (SNP)-genotyping analysis on different populations of Russian (*Acipenser gueldenstaedtii*), Persian (*A. persicus*), and Siberian (*A. baerii*) sturgeon species from the Caspian Sea region (Volga and Ural Rivers), the Azov Sea, and two Siberian rivers. We found that Russian sturgeons from the Volga and Ural Rivers were essentially indistinguishable, but they differed from Russian sturgeons in the Azov Sea, and from Persian and Siberian sturgeons. We identified eight SNPs that were sufficient to distinguish these sturgeon populations with 80% confidence, and allowed the development of markers to distinguish sturgeon species. Finally, on the basis of our SNP data, we propose that the *A. baerii*-like mitochondrial DNA found in some Russian sturgeons from the Caspian Sea arose via an introgression event during the Pleistocene glaciation.

In the present study, the high-throughput genotyping analysis of several sturgeon populations was performed. SNP markers for species identification were defined. The possible explanation of the *baerii*-like mitotype presence in some Russian sturgeons in the Caspian Sea was suggested.

## Introduction

**Figure 1:**
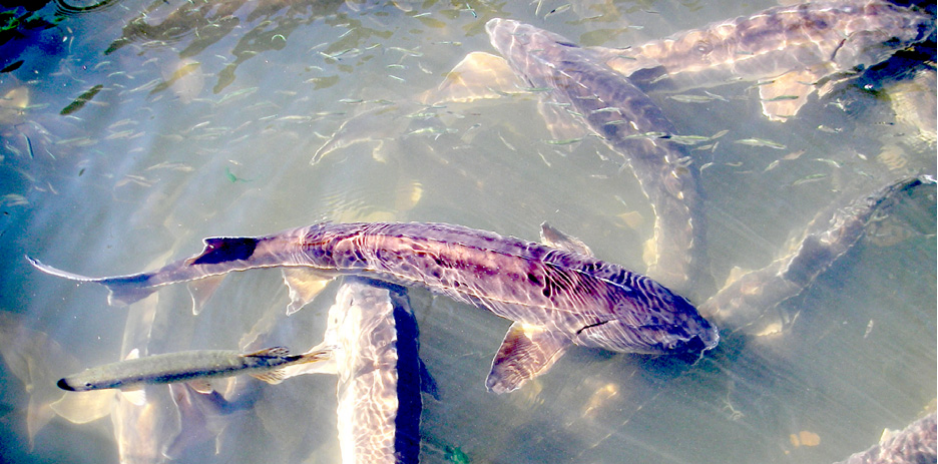
The Russian sturgeon (*Acipenser gueldenstaedtii*) at the Astrakhan fish farm.

The Russian sturgeon (*Acipenser gueldenstaedtii*) is one of the most commercially valuable fish species in the Caspian, Azov, and Black Seas due to its role as a major caviar producer (Vlasenko et al. 35,b; Birstein and Bemis 5) and important aquaculture species (Fig. 1). Three other species are closely related to *A. gueldenstaedtii*: the Persian sturgeon (*A. persicus*), the Adriatic sturgeon (*A. naccarii*), and the Siberian sturgeon (*A. baerii*), the least abundant and hence most valuable species. Together, these four species make up the Ponto-Caspian clade of sturgeons. However, several difficulties have complicated efforts to distinguish between the various species in this clade. *Acipenser persicus* inhabits the Caspian Sea and cannot be readily distinguished from *A. gueldenstaedtii* by mitochondrial markers (Birstein et al. 8; Birstein and Doukakis 6), or by morphological or anatomical differences (Vlasenko et al. 35, b; Ruban et al. 26). Thus, the taxonomic rank of *A. persicus* is disputed, with some authors considering it to be a separate species (Luk'yaneko et al. 16; Putilina 22) and others regarding it to be a subspecies of *A. gueldenstaedtii* (Berg 3; Birstein and Bemis 5; Birstein et al. 8; Birstein and Doukakis 6; Ruban et al. 26). Although the Adriatic sturgeon uniquely inhabits the northern Adriatic Sea, it also morphologically resembles *A. gueldenstaedtii* (Tortonese 34). Unlike its anadromous relatives, the Siberian sturgeon is a freshwater species. Its geographical distribution includes Siberian Rivers and Lake Baikal, and does not overlap with Russian sturgeon. All these species are highly endangered, and are under the control and protection of the Convention for International Trade in Endangered Species (CITES).

The unusual polyphyly of Russian sturgeons’ mitochondrial DNA further complicates their unique identification. Roughly a third of Russian sturgeon specimens from the Caspian Sea have mitochondrial DNA that is similar to that of Siberian sturgeons, which is often referred to as the “*baerii*-like mitotype” (Birstein et al. 8; Jenneckens et al. 11; Rastorguev et al. 23). However, *baerii*-like haplotypes found in the Caspian population of *A. gueldenstaedtii* are different from those of *A. baerii* in one substitution in the highly variable D-loop and can be distinguished by PCR-based test (Mugue et al. 17). Two percent of Russian sturgeons in the Azov Sea have the *baerii*-like mitotype (Timoshkina et al. 33). In contrast, no Persian sturgeons have been found to possess the *baerii*-like mitotype (Mugue et al. 17), supporting that *A. persicus* is genetically isolated entity. Nevertheless, mitochondrial DNA analysis can distinguish only the Siberian sturgeon from among its relatives (Mugue et al. 17).

There are several explanations of the “*baerii*-like mitotype” phenomenon. Some authors propose that the *baerii*-like mitotype arose from farmed *A. baerii* having escaped into the Volga River (Jenneckens et al. 11), whereas others suggest that the *baerii*-like mitotype and the majority *A. gueldenstaedtii* mitotype are ancestral mitotypes for the Siberian, Russian, and Persian sturgeon species (Birstein et al. 9). It is also possible that hybridization between Siberian and Russian sturgeons during the Pleistocene glaciation led to Siberian sturgeon mitochondrial DNA introgression, as considerable changes in the species distributions of other organisms occurred in that period (Hewitt 10).

The identification of nuclear genome markers associated with specific Ponto-Caspian sturgeon species has generally lagged behind due to the polyploidy of the sturgeon genome. It is widely accepted that polyploidization took place several times within the order Acipenseriformes. First whole-genome duplication presumably occurred in the ancestral lineage and led to formation of 2n = 120–140 (“low chromosome number”) group of species. Two independent second rounds of polyploidization were proposed: one in pacific clade, and another one in Atlantic clade of sturgeons (2n = 240–260), leading to formation of two phylogenetically unrelated groups of “high chromosome number” sturgeon species (Birstein et al. 7). Evidence suggests that low chromosome number species are tetraploids with functionally diploid genome, and high chromosome number species are octoploids, with two sets of paralogous tetrasomic loci.

The species relationship between Russian and Persian sturgeons has been addressed recently by restriction site–associated DNA (RAD) sequencing (Baird et al. 1), but the issue remains unresolved (Ogden et al. 20).

Meanwhile, high-throughput single-nucleotide polymorphism (SNP) genotyping provides precise sample assignment to its origin therefore it becomes highly valuable tool against illegal fishing and mislabeling. Effectiveness of such approach was shown for four commercial marine fish species (Nielsen et al. 19).

In this study, we performed high-throughput genotyping analysis of individuals from several populations of Russian, as well as Persian, and Siberian sturgeons to find SNP markers for species identification. The resulting data should aid in sturgeon conservation efforts and help prevent illegal fishing. Additionally, we suggest an explanation of the *baerii*-like mitotype found in some Russian sturgeons in the Caspian Sea region.

## Material and Methods

### RNA isolation and cDNA library construction

A several-month-old Russian sturgeon fingerling from an Astrakhan fish farm (Ltd Astrakhan Fish Breeding Company, also known as “Beluga”) was suspended in RNAlater reagent (Invitrogen/Life Technologies, Carlsbad, CA) for the preparation of a cDNA library. RNA extraction from different tissues (representing total body RNA content) was done with the TRIzol reagent according to the provided RNA extraction protocol (Invitrogen). Recent international FP6 supported project SturSNiP revealed that there is no fixed segregating genetic markers between these species, and all informative markers are frequency dependent due to sheared polymorphism (Ogden et al. 20). Our approach was different from that one of SturSNiP because our aim was to reveal highly polymorphic loci in the Caspian population of Russian sturgeon; therefore, we collected transcriptome information from a single individual rather than from pooled samples from different populations of different species. There was twofold rationale behind taking one fingerling for transcriptome analysis; first, we attempted to analyze the entire mRNA repertoire, so we needed artificially propagated fingerling to be sacrificed and the diversity of tissues to be sampled, and second, the data of allelic composition from one individual allowed us to avoid problem of paralogous (resulted from previous whole-genome duplication) loci and to discriminate paralogous loci from true polymorphic sites (see below). cDNA was prepared from total RNA by a M-MLV reverse transcriptase approach (Schmidt and Mueller 28) using the MINT cDNA synthesis kit (Evrogen, catalog #SK001). The resulting “SMART” cDNA was amplified and normalized (reducing the levels of highly abundant transcripts), using the duplex-specific nuclease (DSN) normalization method (Zhulidov et al. 37).

### Transcriptome sequencing and mapping

Approximately 5 μg of nonnormalized cDNA and 10 μg of normalized cDNA were used for SOLiD library construction and sequencing using the SOLiD™ 4.0 system (Life Technologies) according to the standard protocol (SOLiD User Guide). Reads from the normalized and nonnormalized libraries were mapped against the sequence of Russian sturgeon mitochondrial DNA (NCBI accession number NC_012576.1), and all reads apparently derived from mitochondrial DNA were excluded from further mapping. The remaining reads were mapped against the sturgeon EST database from GenBank using the Bowtie software package (Langmead et al. 12). An EST-derived reference database was created using sequences bearing the “Acipenser” tag in the “species name” field (6219 sequences in total), and was then used for mapping. SNP calling was performed using the SAMtools package (Li et al. 14; Li 13). Mismatches between mapped reads were catalogued in VCF files, and heterozygous positions were then identified with a custom Perl script. SNP positions were sorted by coverage, and those that were covered by more than 100 reads were selected for the further analysis.

### Sturgeon samples and genotyping

Sturgeon samples were obtained from The Russian National Collection of Reference Genetic Materials (RNCRGM), developed by the Russian CITES scientific authority on sturgeon fishes and maintained by VNIRO. Population samples of Caspian basin Russian sturgeons originated from the Volga and Ural Rivers (14 specimens each), with five additional specimens of Russian sturgeons originating from the Azov Sea; 28 Persian sturgeon specimens originated from the northern Caspian Sea; and five Siberian sturgeon specimens originated from the Lena and Irtysh rivers. DNA was isolated using the Wizard^®^ SV 96 Genomic DNA Purification System (Promega, Fitchburg, WI).

Custom GoldenGate Genotyping Panel was constructed in Illumina Inc., San Diego, CA using loci, selected from SNP calling procedure described above (Table S1). The GoldenGate assay was performed using the Illumina GoldenGate platform according to the manufacturer's instructions and analyzed by the genotyping module of Illumina's Genome Studio data analysis software (Illumina Inc.). Loci for which all specimens showed the same genotype or genotypes could not be distinguished by this software, and were excluded from further analysis.

### Genotyping data analysis

An UPGMA (unweighted pair group method with arithmetic mean) tree of sturgeon populations was constructed using the POPTREE2 software (Takezaki et al. 32), with *F*_ST_ distances according to Latter (101). We used the Whichloci program to identify SNP loci with the highest statistical power for population assignments (Banks et al. 2). For the Whichloci analysis, we generated four simulated datasets by random resampling the SNP loci (three with sample size 100, one with sample size 500), and we then ran Whichloci on each dataset and chose loci that were present in the outcomes from all four simulated datasets. Population structure was inferred using the STRUCTURE software (Pritchard et al. 21), with parameters “prior sample location information” and “allele frequency model correlated among populations” included in the admixture model. *F*_ST_ statistics – the effect of subpopulations (**s**) compared to the total population (**t**) – were determined using the GenePop web service (http://genepop.curtin.edu.au/; Raymond and Rousset 24; Rousset 25), with the “*F*_ST_ and other correlation” options.

## Results and Discussion

### RNA-Seq analysis of the sturgeon transcriptome

Transcriptome data for the Russian sturgeon fingerling were generated from approximately 250 million 50-base reads of a normalized cDNA library and 80 million reads of a nonnormalized cDNA library using the SOLiD (Life Technology) platform. After removing mitochondrial DNA reads, approximately 1.7 million reads from the normalized library and 3 million reads from the nonnormalized library mapped to the GenBank sturgeon expressed sequence tag (EST) database (Table 1; note that the reduced number of mapped reads for the normalized library was expected due to the removal of ribosomal and tRNA transcripts during the normalization process; 0.3% [821,892 of 252,186,716] of normalized library reads and 4.6% [3,624,935 of 79,030,219] of control library reads were mapped on ribosomal database). From 6219 totals, 4300 ESTs were covered by at least one read. The overall low numbers of mapped reads could be explained by the fact that the database contains mainly *A. sinensis* and *A. transmontanus* ESTs, which are distantly related to *A. gueldenstaedtii*.

**Table 1:** SOLiD read mapping and SNP calling statistics for two *Acipenser gueldenstaedtii *cDNA libraries

	Normalized cDNA library	Nonnormalized cDNA library
Total reads	252,186,716	79,030,219
Mapped reads	1,787,162	3,159,679
Total SNPs called	1,014,088	755,147
Heterozygous SNP	50,124	30,682
SNPs (coverage ≥10)	611,971	415,411
Heterozygous (coverage ≥10)	45,645	27,143
SNPs (coverage ≥100)	181,683	138,058
Heterozygous (coverage ≥100)	17,765	9812

Read mapping was performed using the sturgeon EST database as a reference. SNP calling was done with default SNP caller settings (using SAMtools) and with minimum coverage depths of 10 and 100.

### SNP discovery for sturgeon genotyping and population identification

Several thousand SNP candidates were identified by screening heterozygous positions using the following criteria of read sequence quality, read mapping quality, and quality score at the mismatch position. Additionally, we required that the presumptive SNPs have allele ratios close to 3:1 based on the following assumptions: “high chromosome number” sturgeons, including three species in our study, have two sets of paralogous tetrasomic loci (total eight similar genomic regions, segregated as two tetrasomic loci), special precaution was applied to avoid erroneous considering alternatively fixed paralogous loci as a polymorphic locus. Thus, alleles with 1:1 ratios would likely represent divergent paralogous loci which would not be polymorphic across individuals in a population. Therefore, we sought to identify genotypes where one tetrasomic set of alleles was heterozygous and the other locus (paralog) was homozygous. Allelic ratio 3:1 would likely represent situation when one paralog is fixed (i.e., AAAA) and the other one is polymorphic (AABB). Total 384 of the best-scoring mismatch positions were chosen for a GoldenGate custom SNP panel (Illumina) for sturgeon genotyping (Table S1). The GoldenGate assay revealed that 123 of the 384 variable loci were successfully genotyped; the others appeared homozygous or heterozygous across all individuals (fixed at the same or alternative alleles at paralogous loci) and were excluded from further analysis. Loci that appeared heterozygous across all individuals most likely reflected the presence of a paralogous locus, creating the appearance of two different alleles.

### Analysis of sturgeon population structure

To perform a population structure analysis, genotype calls were filtered from the Illumina Genome Studio report into the STRUCTURE (Pritchard et al. 21) and GenePop (Raymond and Rousset 24) programs. The Whichloci program (Banks et al. 2) guided the choices of the most informative loci for population assignment. We detected few genetic differences at the 123 diagnostic positions between the Volga River and Ural River populations of Russian sturgeons. Eight of the most informative SNP loci were sufficient to assign specimens to the Caspian Russian (Volga and Ural River) populations, Russian Azov Sea population, and Persian populations with 80% accuracy. A total of 12 loci were sufficient to make these population assignments with 90% accuracy (Table 2).

**Table 2:** DNA sequences of the 12 SNP candidate markers used for sturgeon population assignment

Marker name	DNA sequence
EV824350.93	TTCGATACGATAAGCCGCAATGTCTGCAGGAACACTGGCTAAACCCGCAATGCGCGGTCT[A/G] CTCGGTAAACGTCTACGATTCCACCTGGCTGTTGCTTTCACGCTGTCTTTGGCTGCAGCA
ES698489.358	ATTTTAAAGTGGARCCCTTTGTTGTTGATTCCTGGTTTAAATTTCCAGCACCTTCACAAA[A/T]TC CAGTAAGAAAGACATGTGAAGTAGACMSTGGCCGTGTATTTCAGATGTGCTCTGATAA
DR977078.72	TTGCCCAGGTCTTGAACAATATGCTATCAAGAAGTTTGCAGAGGCTTTTGAAGCCATTCC[A/G]C GTGCTCTTGCAGAGAACTCTGGTGTCAAGGGCAATGAACTTATCTCCAAACTGTATGCT
DR976209.314	GAACAGTGCACTCCACAAGAATTGTTTTTAGCCGTATAACTTCAAGAACAGAAAACTAAG[T/C] TGCATTATGAGTGGAGGAAGACAGATGACTGCGCTACTGTACACCCCTTTTACAACAASA
DR977006.239	CTCTTTCACCTTTGAGCTTCGTGATACTGGCAGATATGGATTCCTCCTTCCAGAGTCGCA[A/G]A TCAGGCCAACTTGCCAAGAAACAATGCTGGCAGTCAAATACATTGCCAAACATGTTCAG
EV824380.474	CCAAGGGCAATGTACCGCAACTTCTGGAGCCCATCCCATACGAGTTCATGGCGTAAAGCA[T/C] GGTCAAGCCAGAATAAAGTCCTGTTTTCAACAAAAAAAAAAAAAAAAAAAAANAAANNNA
DR976578.395	TTTCCCCATCAATGTGGTCGTTCAGGAAACTGGGTCTCTGGTGGAAATCAGGAATTTCTT[A/G]G GAGAAAAGTACATCCGCAGAGTACGCATGAGGCAGGGTGTGACCTGTAATGTTTCCCAG
DR975536.188	TAGAAGAGGGCGCCTGACCAAGCACACCAAGTTYGTTCGGGACATGATCCGCGAGGTGTG[T/C] GGCTTCGCCCCCTACGAGAGACGCGCCATGGAGCTGCTGAAGGTCTCCAAGGATAAGCGM
DR977561.396	GAAGTTTGCCTGCAATGGGACTGTGATTGAGCATCCAGAATATGGTGAAGTGGTTCAGCT[A/G] CAGGGTGATCAGCGCAAGAATATCTGCCAGTTCCTTACTGAGATTGGCTTGGCTAAGGAG
ES698197.350	AACAAGCCAAGGACAAGCCCCAGGAGAAACCCAAATAACATGGCAAGAGRACTAACCATC[A/G] CTTACAAGAGAAGTTTCAATCTCCGACACAAGCTTCCTGTCTGGGATTTCATTTTCMTTT
DR974886.648	TTGGTACCGTGTATCTCTCTGCTCTGGCTTTATAATGATGGGTGTCACCGTATATGAAGG[A/G]G GCACCTGGTAATGCASATGGTAAACCAATCAGTAATGTAAAGAATTGGATGCAATAAAG
ES697699.316	GAGAAGATYGACCTGAAGTTCAACCACCTCCAAGTTCGGACACGGACGCTTCCAGACCGC[T/C] GAAGAGAAGAAGGCGTTCATGGGACCACTCAAAAAGGACCGAATCCTCAAGGAGGAGACT

Each polymorphic position is shown in square brackets. Sequence names consist of the GenBank accession number of the candidate EST sequence, with the SNP position within the EST given after the period.

**Figure 2:**
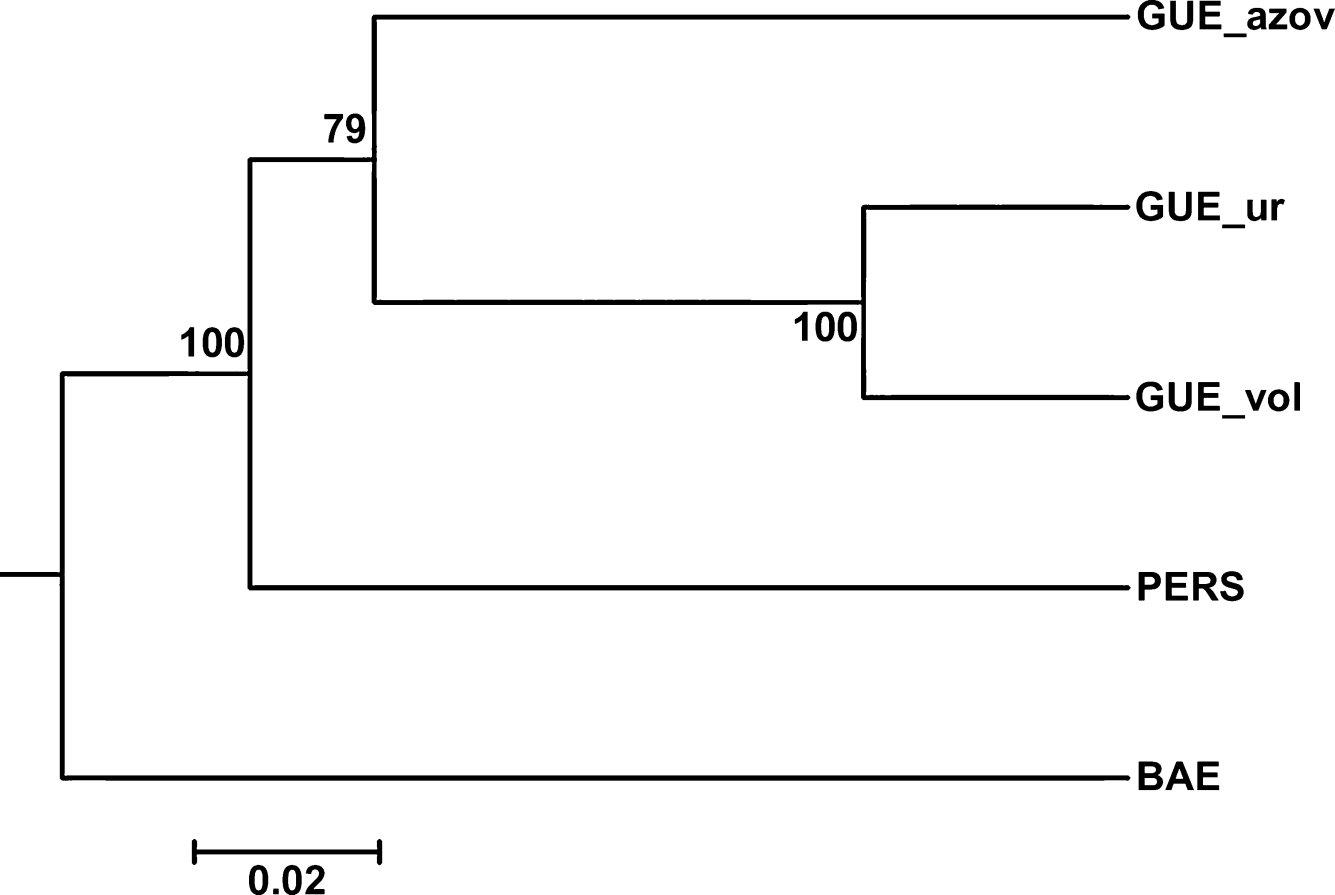
UPGMA tree showing the relationships of sturgeon populations (POPTREE2; Takezaki et al. 32). Genetic distances (*F*_ST_) between the populations (Latter 101) were estimated using allele frequency data for 123 loci. Bootstrap values are indicated next to corresponding nodes. GUE_azov, *Acipenser gueldenstaedtii* from the Azov Sea; GUE_ur, *A. gueldenstaedtii* from the Ural River; GUE_vol, *A*. *gueldenstaedtii* from the Volga River; PERS,* A*. *persicus*; BAE,* A. baerii*. (The Ural and Volga River sturgeons are considered to be representative of Caspian Sea Russian sturgeons.)

**Figure 3:**
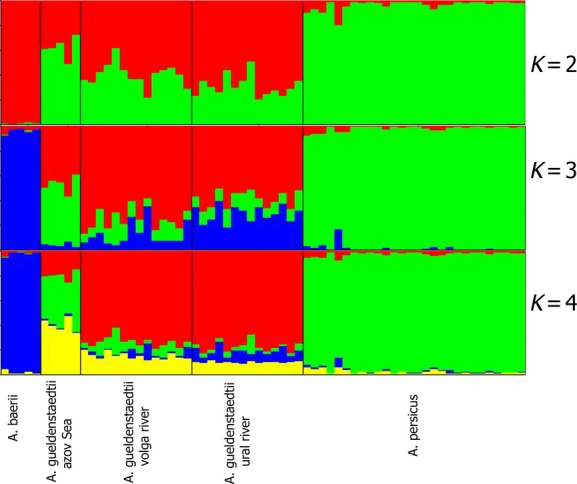
Population structure analysis of various sturgeon samples using the STRUCTURE software. Three runs of STRUCTURE with the cluster number (K) set to two, three, or four are shown. Siberian (*Acipenser baerii*) and Persian (*A. persicus*) sturgeons form their own distinct clusters, whereas all three Russian sturgeon populations have mixed origins.

Using the allele frequencies within each population for all 123 polymorphic SNP loci (taken from the Genome Studio report), we constructed a population tree (Fig. 2). As expected, the Siberian sturgeons appeared more distant from the other species of the Ponto-Caspian clade. Our data also indicated that the Persian sturgeon was separated from all three samples of the Russian sturgeon, suggesting that *A. persicus* is genetically different from all *A. gueldenstaedtii* populations. These data do not support an opinion of Ruban et al. (26) that *A. persicus* is an ecological morph within the Caspian Sea population of Russian sturgeon, but not a valid species. The population assignment analysis (STRUCTURE) also supported the genetic separation of the Persian sturgeon, as a homogeneous cluster consisted exclusively of *A. persicus* specimens (Fig. 3). The Siberian and Persian populations were clearly separated into two distinct groups, whereas the Russian sturgeons appeared as intermediate and shared population structure elements of both the Siberian and Persian sturgeons. This is likely due to nature of our SNP loci when polymorphic loci were obtained from Russian sturgeon specimen.

### Siberian sturgeon introgression into Caspian Russian sturgeon population during the Pleistocene glaciation

Frequency of several SNPs of the Caspian (Volga and Ural Rivers) populations of Russian sturgeon shows an intermediate value compared to those in Siberian and Persicus populations, indicating that Caspian population of the Russian sturgeon retains traces of ancient introgression of Siberian sturgeon into ancestral Caspian population of the Russian sturgeon during the Pleistocene glaciation. These data support the hypothesis that the *baerii*-like mitotype is a result of this ancient introgression. The climatic oscillations during the glaciation caused great changes in species distribution, and considerable evidence suggests that other organisms dispersed to new locations (Hewitt 10). An investigation of 41 North American fish species concluded that the Pleistocene glaciation had a great impact on fish population structure (Bernatchez and Wilson 4). The presence of distinct mitochondrial DNA sequences within a species has been explained as a consequence of intraspecific lineage reorganization, whereby the dispersed lineages of an ancestral set of refugees reunite in one formerly glaciated region (Taberlet and Bouvet 31; Santucci et al. 27).

Notably, the hypothesis of the interspecific hybridization is required to explain abundance of the *baerii*-like mitotype and nuclear markers in the Caspian population of *A. gueldenstaedtii*. This scenario is supported by evidence that hybridization between Russian and Siberian sturgeons leads to the formation of fertile hybrids (Jenneckens et al. 11), which are widely used in aquaculture. Considering that the glaciation allowed the Siberian sturgeons into the Caspian Sea, there may have been nothing to prevent their hybridization with Caspian sturgeons. Previously published data that “*baerii*-like” mitotypes are present in the Caspian Russian sturgeon population and absent from the Persian population are consistent with this notion (Mugue et al. 17).

## Conclusions

In this study, we discovered SNP markers for distinguishing between Caspian and Azov Sea populations of Russian sturgeons, as well as between Russian, Persian, and Siberian sturgeons. These data allow the development of a genetic test that can be used for law enforcement in sturgeon fishery, aquaculture industry, and trading, as well as in conservation efforts for endangered sturgeon species. Finally, our SNP analysis supported the idea that the mitochondrial DNA polyphyly of Russian sturgeons in the Caspian Sea resulted from an introgression event during the Pleistocene glaciation.

## Data Accessibility

The SOLiD sequence data in .csfasta + .quality formats, mapped data in SAM/BAM formats, and SNP variants in .vcf format are available on request.
